# *In vitro* significance of SOCS-3 and SOCS-4 and potential mechanistic links to wound healing

**DOI:** 10.1038/s41598-017-06886-6

**Published:** 2017-07-27

**Authors:** Yi Feng, Andrew J. Sanders, Liam D. Morgan, Sioned Owen, Fiona Ruge, Keith G. Harding, Wen G. Jiang

**Affiliations:** 1 Cardiff China Medical Research Collaborative, Cardiff University School of Medicine, Cardiff University, Cardiff, CF14 4XN UK; 2 Wound Healing Research Unit, Cardiff University School of Medicine, Cardiff University, Cardiff, CF14 4XN UK

## Abstract

Wound healing and the management of chronic wounds represent a significant burden on the NHS. Members of the suppressor of cytokine signalling (SOCS) family have been implicated in the regulation of a range of cellular processes. The current study aims to explore the importance of SOCS-3 and SOCS-4 in regulating cellular traits associated with wound healing. SOCS-3 over-expression and SOCS-4 knockdown mutant lines were generated and verified using q-PCR and western blotting in human keratinocytes (HaCaT) and endothelial cells (HECV). Over-expression of SOCS-3 resulted in a significantly reduced proliferative rate in HaCaT keratinocytes and also enhanced the tubule formation capacity of HECV cells. SOCS-4 knockdown significantly reduced HaCaT migration and HECV cell tubule formation. Suppression of SOCS-4 influenced the responsiveness of HaCaT and HECV cells to EGF and TGFβ and resulted in a dysregulation of phospho-protein expression in HaCaT cells. SOCS-3 and SOCS-4 appear to play regulatory roles in a number of keratinocyte and endothelial cellular traits associated with the wound healing process and may also be able to regulate the responsiveness of these cells to EGF and TGFβ. This implies a potential regulatory role in the wound healing process and, thus highlights their potential as novel therapies.

## Introduction

Chronic wounds are defined as wounds that fail to follow the orderly and timely reparative process seen in normal healing, which in turn disrupts the anatomic and functional integrity of the wound site^1^. Chronic wounds have been considered to be a significant medical burden to the general public healthcare system both in terms of cost and resources^2^.

There are many types of chronic wounds with the majority being categorised into four subtypes, pressure ulcers, arterial and venous ulcers as well as diabetic ulcers, which have diverse aetiologies^3, 4^. Venous ulceration is one of the most common lower extremity ulcerations, requiring long-term care, displaying high recurrence rates and accounting for a substantial amount of healthcare budgets worldwide^5, 6^. Diabetic foot ulcers also contribute to the significant medical burden, not only through the considerable expenditure on diabetic foot and amputation care, but also through the negative impact associated with high morbidity and mortality rates^7^. Many medical treatment strategies and guidelines have been developed and are evolving to make the management of chronic wounds more efficient and cost effective, however, the implementation of such strategies still remains challenging and are limited to certain types of disease^6, 7^. To date, the molecular mechanisms involved in chronic wound formation still remain unclear due to the complexity of the wound healing process and the diverse aetiologies of different types of chronic wounds. Therefore, investigations into the cellular impact of key molecules in fundamental cell types involved in the wound healing process and further examination of potential upstream and downstream mechanisms involved, are essential for the generation of bio- or prognostic markers and new therapeutic strategies to combat and aid in the management of chronic wounds.

Wound healing is a sophisticated biological process in which a variety of cell types synergistically coordinate to regenerate functional new skin tissue. Numerous cytokines and growth factors are derived from these cells and regulate signalling cascades which contribute to wound closure. However, dysregulation of cytokine signalling can lead to abnormalities in cellular functions, extended healing times and impairment of the normal healing process, finally leading to non-healing chronicity^8^. Suppressor of cytokine signalling (SOCS) proteins have been recognised as classic cytokine-inducible negative feedback inhibitors^9^. Once synthesised, SOCS proteins act to target and deactivate the Janus kinase/signal transducers and activators of transcription (JAK/STAT) pathway, a common pathway utilised in cytokine signalling. This is via i) inhibition of JAK tyrosine kinase activity through binding to the activated JAK protein; ii) competing with STAT for the cytokine receptor docking site; iii) promoting the proteasomal degradation of SOCS-target protein complex^10^, ensuring that JAK/STAT cytokine signalling is only maintained for an appropriate amount of time. Hence, SOCS can act as an ‘automatic switch’ to control the homeostasis of activated cytokine or growth factor signalling. SOCS proteins are a family of intracellular proteins containing eight members^11^. Several members of the SOCS family have been extensively studied in different areas of research and have been discovered to be able to regulate a wide variety of cytokines and growth factors which play key roles in the wound healing process^12^.

SOCS-3 is one of the most extensively studied SOCS family members. A previous *in vivo* study has indicated that knockdown of SOCS-3 in epithelial basal keratinocytes contributes to severe skin inflammation, indicating an important role in skin homeostasis^13^. However, over-expression of SOCS-3 has also been shown to lead to impaired wound healing due to the suppression of keratinocyte proliferation and migration in a transgenic mouse model^14^. Moreover, in addition to disrupting acute wound healing, mice over-expressing SOCS-3 also exhibit a prolonged inflammation phenotype that resembled characteristics of chronic wounds^15^. Taken together, these studies demonstrate the important regulatory role of SOCS-3 in the wound healing process. In contrast to SOCS-3, SOCS-4 is a poorly investigated member of this family of proteins. Studies have indicated that SOCS-4 plays regulatory roles in inflammation caused by influenza and parasite infection^16, 17^. An additional study has provided evidence to suggest that SOCS-4 may hold the potential to regulate hypoxia inducible factor (HIF)−1α, a mediator involved in the adaptation to hypoxia^18^. Since inflammation and hypoxia are involved in wound healing, SOCS-4 may also have a regulatory role within the wound healing process.

Previous work within our laboratories has explored the expression of SOCS 1–7 in a cohort of chronic healing and non-healing wounds and demonstrated an upregulation of SOCS-3 and SOCS-4 transcript expression in non-healing chronic wounds, further suggesting the potential for these members to influence the healing process^19^. The current study explores the impact of SOCS-4 knockdown and SOCS-3 over-expression on keratinocyte and endothelial cell function to further characterise their potential to impact on traits associated with the healing process.

## Results

### Expression profile of SOCS-1 – SOCS-7 in HaCaT and HECV cell lines

**Figure 1:**
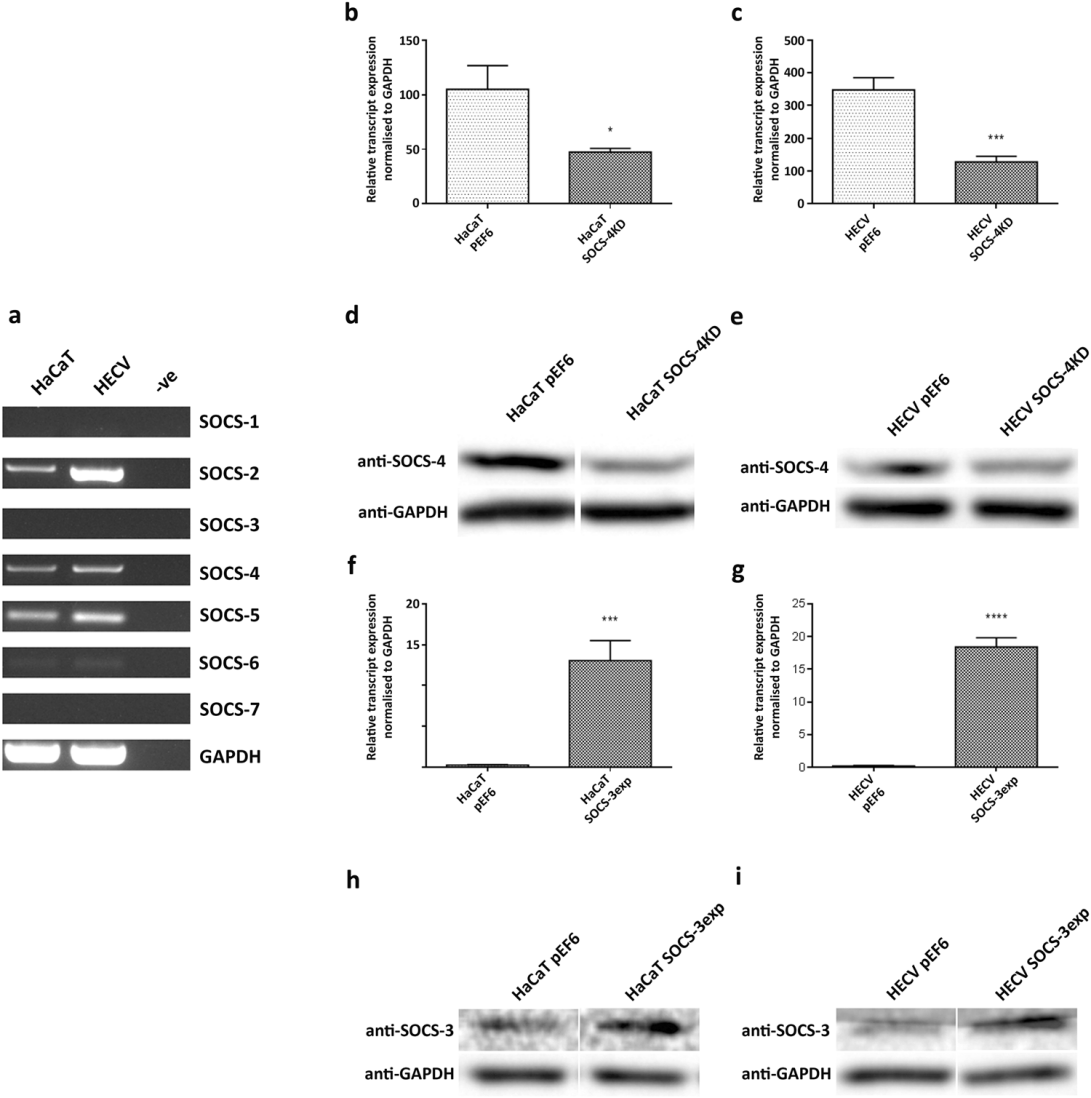
Expression profile and generation of SOCS-3 over-expression and SOCS-4 knockdown models. (**a**) RT-PCR screening of HaCaT and HECV cell lines for SOCS-1 (108 bp), SOCS-2 (461 bp), SOCS-3 (521 bp) SOCS-4 (541 bp), SOCS-5 (117 bp), SOCS-6 (131 bp), SOCS-7 (590 bp) and GAPDH (475 bp), negative control (-ve) represents amplification of molecular biology grade water instead of cDNA. Verification of SOCS-4 transcript knockdown was assessed and confirmed using q-PCR in (**b**) HaCaT and (**c**) HECV cell lines. Protein knockdown in (**d**) HaCaT and (**e**) HECV cells were verified using western blot analysis. Verification of SOCS-3 over-expression at transcript level was confirmed using q-PCR in (**f**) HaCaT and (**g**) HECV cell lines. Protein knockdown was verified in (**h**) HaCaT and (**i**) HECV cells using western blot analysis. RT-PCR and western blot data represents cropped images, full length gels and blots are included in supplementary information. Representative data of a minimum of three independent repeats shown, error bars represent standard deviation, * represents p < 0.05, *** represents p < 0.001, **** represents p < 0.0001.

The expression profile of SOCS-1 to SOCS-7 was explored in human HaCaT keratinocytes and HECV endothelial cell lines using RT-PCR (Fig. 1a). RT-PCR demonstrated that SOCS-2, SOCS-4, SOCS-5 and SOCS-6 were expressed in both HaCaT and HECV cell lines. Furthermore, the transcript levels of these four SOCS family members exhibited higher expression in HECV cells to that in HaCaT cells, though expression of SOCS-6 was weak in comparison to SOCS-2, -4 and -5. No detectable expression of SOCS-1, SOCS-3 and SOCS-7 were observed in either HaCaT or HECV cells.

### Generation of HaCaT and HECV SOCS-4 knockdown and SOCS-3 over-expression models

To further explore the significance of SOCS-3 and SOCS-4, over-expression or knockdown models were established in HaCaT keratinocytes and HECV endothelial cells through transfection with either SOCS-3 coding sequences or ribozyme transgenes specifically targeted to SOCS-4. Following transfection and selection of the cell lines, SOCS-3 over-expression or SOCS-4 knockdown was assessed at the transcript and protein level using q-PCR and western blotting respectively. Transfection of both HaCaT and HECV cells with the SOCS-4 ribozyme transgene significantly reduced transcript expression of SOCS-4 in comparison to the HaCaT or HECV pEF6 plasmid controls (Fig. 1b,c; p < 0.05 or p < 0.001, respectively). Similarly, SOCS-4 protein knockdown was observed in both cell lines in comparison to the pEF6 plasmid controls with a 48% and 22% decrease in SOCS-4 expression observed in HaCaT and HECV cells respectively following transfection with the SOCS-4 ribozyme transgene (Fig. 1d,e). Transfection of HaCaT and HECV cells with the SOCS-3 over-expression plasmid also significantly enhanced transcript levels of SOCS-3 in comparison to controls transfected with the pEF6 plasmid alone (Fig. 1f,g; p < 0.001 or p < 0.0001, respectively) and resulted in an obvious increase in SOCS-3 protein expression, demonstrating 40% and 48% increases in protein levels compared to HaCaT and HECV pEF6 controls respectively (Fig. 1h,i).

### Impact of SOCS-3 over-expression and SOCS-4 knockdown on cell proliferation and attachment

**Figure 2:**
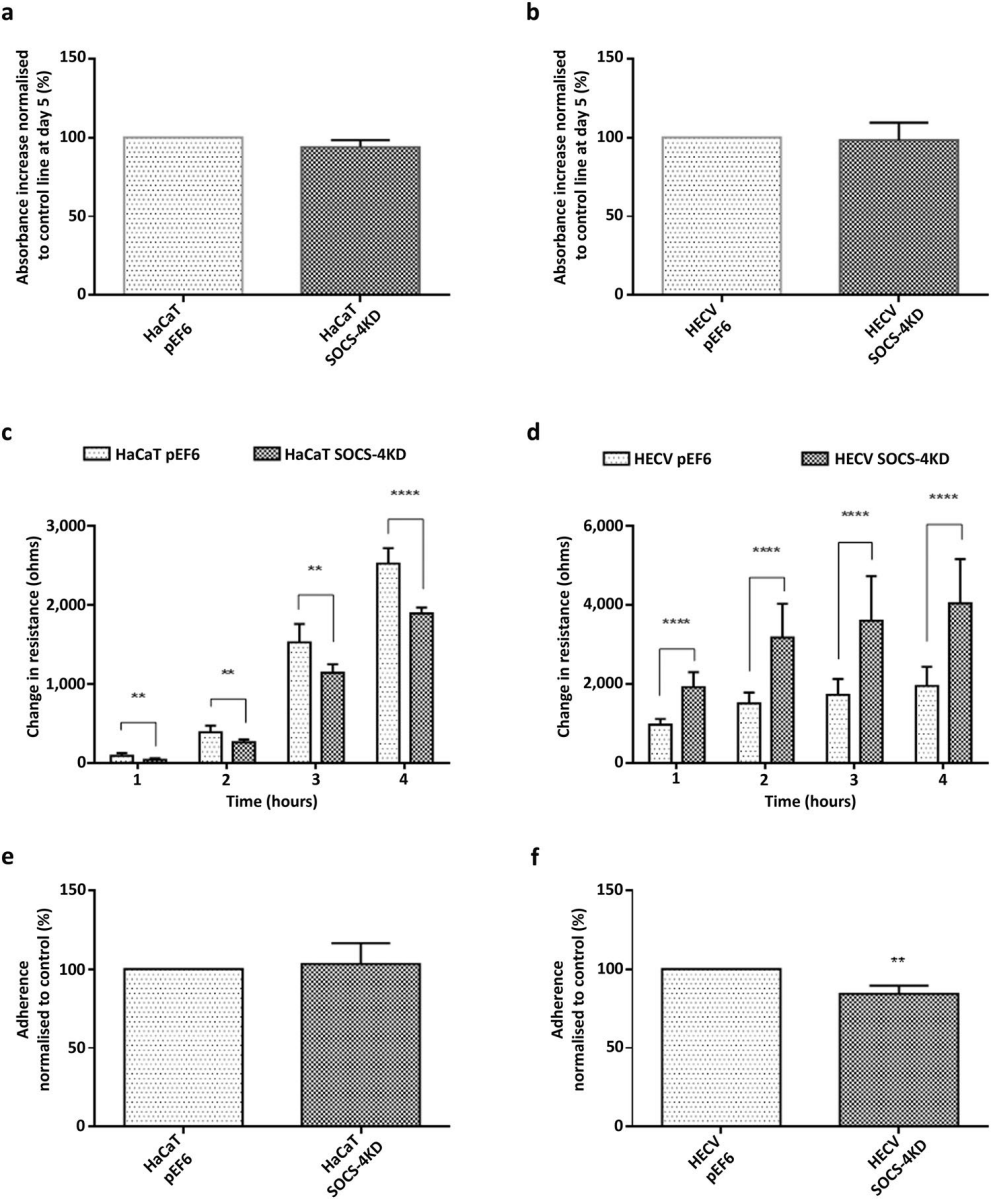
Impact of SOCS-4 knockdown on cell proliferation and attachment. Five day proliferation rates of (**a**) HaCaT and (**b**) HECV cells following SOCS-4 knockdown, percentage respective pEF6 control of at least three independent repeats are shown, error bars represent the standard error of the mean. Cell attachment was assessed using an ECIS based method over 4 hours post seeding in (**c**) HaCaT and (**d**) HECV. Data, representative of at least three independent repeats shows mean change in resistance as detected by the ECIS system and was used to indicate cell attachment to the electrode, error bars represent standard deviation. A Matrigel matrix adhesion assay was also implemented to examine Matrigel attachment of (**e**) HaCaT and (**f**) HECV cells following SOCS-4 knockdown, mean percentage respective pEF6 control of at least three independent repeats are shown and error bars represent the standard error of the mean. ** represents p < 0.01, *** represents p < 0.001 and **** represents p < 0.0001.

Knockdown of SOCS-4 did not impact on the 5 day proliferative rates of HaCaT (Fig. 2a) or HECV (Fig. 2b) cells and no significant differences were seen between SOCS-4 knockdown cells and their respective pEF6 plasmid control cell lines (p > 0.05). Similarly, no differences in 3 day growth rates were seen between SOCS-4 knockdown and the respective pEF6 control in either HaCaT or HECV cell lines (p > 0.05, data not shown). The impact on cell attachment and adhesion following SOCS-4 knockdown was assessed in both cell lines using an ECIS based attachment model (Fig. 2c,d) and a Matrigel adhesion model (Fig. 2e,f). Conflicting results were observed between the assays. ECIS analysis indicated that SOCS-4 knockdown reduced HaCaT cell attachment, as detected by change in resistance, in comparison to pEF6 control HaCaT cells at each time point (Fig. 2c; 1 hour, 2 hour, 3 hour, p < 0.01; 4 hour p < 0.0001) whereas the opposite was seen in the HECV model where SOCS-4 knockdown enhanced cell attachment at each tested time point in comparison to HECV pEF6 control cells (Fig. 2d; p < 0.0001 at each time point). Utilising a different model, exploring cell attachment to a Matrigel artificial basement membrane, no significant differences in attachment were seen between HaCaT SOCS-4 knockdown and the pEF6 control line (Fig. 2e; p > 0.05) though a significant decrease in Matrigel attachment was seen following SOCS-4 knockdown in HECV cells compared to the HECV pEF6 control (Fig. 2f; p < 0.01).

**Figure 3:**
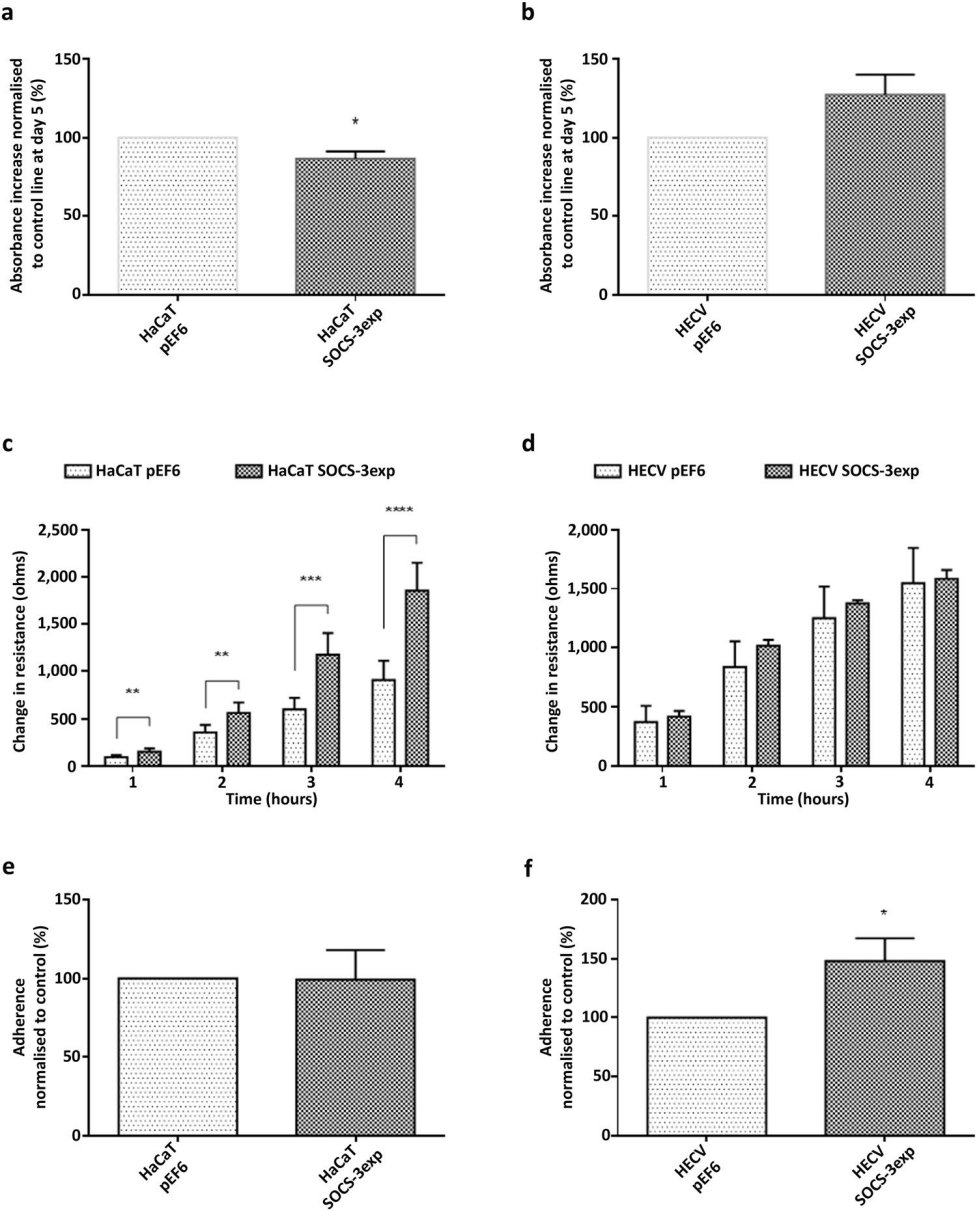
Impact of SOCS-3 over-expression on cell proliferation and attachment. Five day proliferation rates of (**a**) HaCaT and (**b**) HECV cells following SOCS-3 over-expression, percentage respective pEF6 control of at least three independent repeats are shown and error bars represent the standard error of the mean. Cell attachment was assessed using an ECIS based method over 4 hours post seeding in (**c**) HaCaT and (**d**) HECV. Data, representative of at least three independent repeats shown, mean change in resistance, detected by the ECIS system, was used to indicate cell attachment to the electrode and error bars represent standard deviation. A Matrigel matrix adhesion assay was also implemented to examine Matrigel attachment of (**e**) HaCaT and (**f**) HECV cells following SOCS-3 over-expression, mean percentage respective pEF6 controls of at least three independent repeats are shown and error bars represent the standard error of the mean. * represents p < 0.05, ** represents p < 0.01, *** represents p < 0.001 and **** represents p < 0.0001.

Over-expression of SOCS-3 decreased the 5 day proliferative rate of HaCaT cells in comparison to the pEF6 plasmid control cells (Fig. 3a; p < 0.05) but did not significantly affect HECV cells (Fig. 3b; p > 0.05). No significant alterations were seen between SOCS-3 over-expression and respective pEF6 controls over a 3 day incubation period in either the HaCaT or HECV cell models (p > 0.05; data not shown). Using the ECIS based attachment model, an opposite trend to SOCS-4 knockdown was seen when SOCS-3 was over-expressed in HaCaT cells. SOCS-3 over-expression brought about an enhanced rate of cell attachment in comparison to HaCaT pEF6 control cells (Fig. 3c; 1 hour, 2 hour p < 0.01; 3 hour p < 0.001, 4 hour p < 0.0001). No significant effects on ECIS based attachment were seen between HECV SOCS-3 over-expression cells and HECV pEF6 control cells at all time points (Fig. 3d; p > 0.05 at each time point). In relation to attachment to Matrigel, SOCS-3 over-expression did not impact on HaCaT cell Matrigel attachment, with no significant differences observed between HaCaT SOCS-3 over-expression and HaCaT pEF6 cells (Fig. 3e; p > 0.05), but did significantly enhance HECV attachment in comparison to HECV pEF6 controls (Fig. 3f; p < 0.05).

### Impact of SOCS-3 over-expression and SOCS-4 knockdown on cellular migration and angiogenic potential

**Figure 4:**
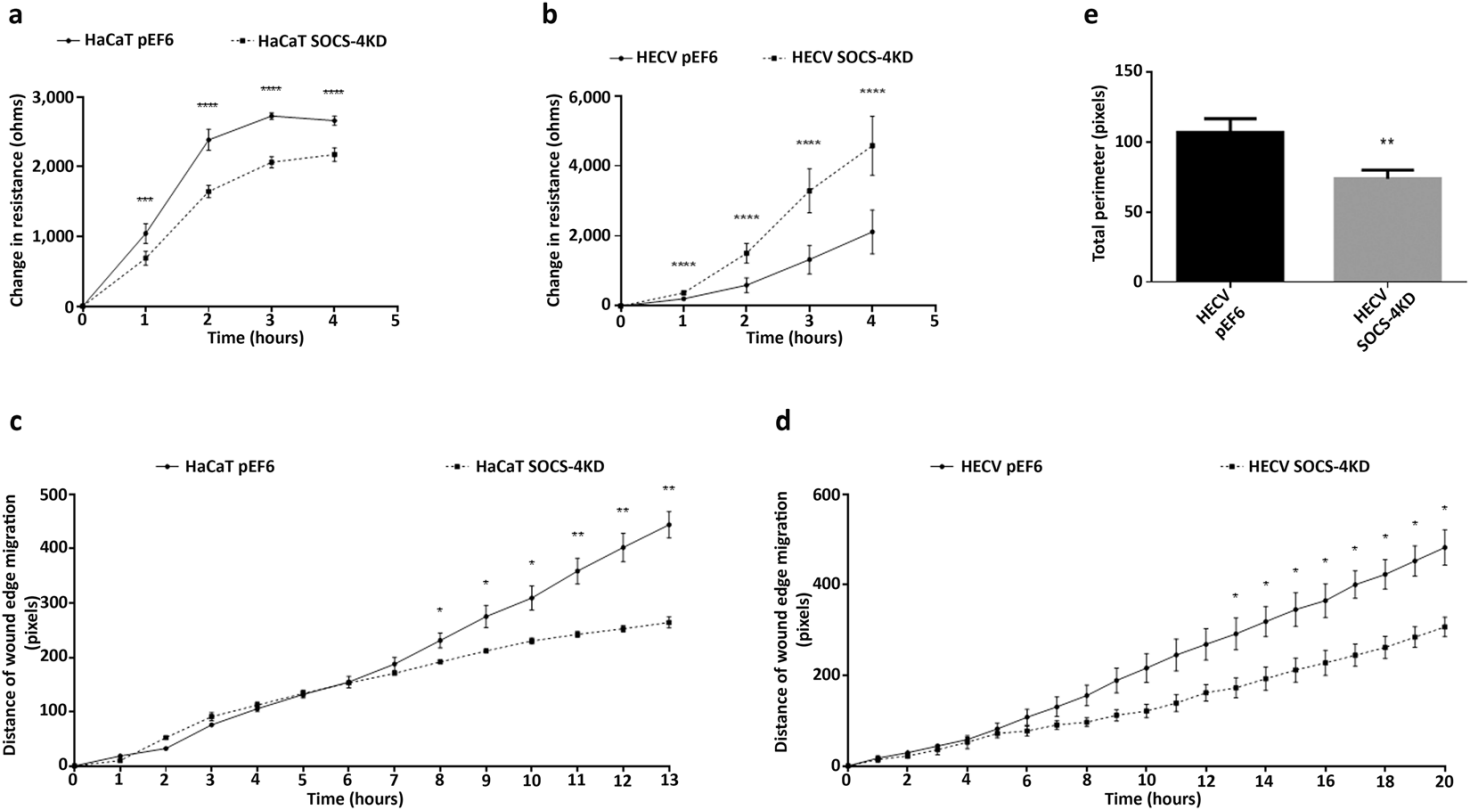
Impact of SOCS-4 knockdown on cellular migration and tubule formation. Cellular migration was assessed using an ECIS model in (**a**) HaCaT and (**b**) HECV cells following SOCS-4 knockdown. Mean change in resistance following monolayer wounding was detected by the ECIS system and was used to quantify cell migration. Cellular migration was also explored using a conventional scratch wound assay, monitored by the EVOS system, in (**c**) HaCaT and (**d**) HECV cells following SOCS-4 knockdown. Angiogenic potential was assessed, following SOCS-4 knockdown in HECV cells using a Matrigel tubule formation assay (**e**). Data shown is representative of a minimum of three repeats, error bars represent standard deviation (a/b/e) and standard error of the mean (c/d), * represents p < 0.05, ** represents p < 0.01, *** represents p < 0.001 and **** represents p < 0.0001.

SOCS-4 knockdown was seen to significantly decrease the migratory rate of HaCaT cells, in comparison to HaCaT pEF6 control cells, in an ECIS based migration assay (Fig. 4a). Significant differences were seen between HaCaT SOCS-4 knockdown and HaCaT pEF6 controls at all time points compared (1 hour p < 0.001, 2 hour, 3 hour, 4 hour p < 0.0001). However, SOCS-4 knockdown in the HECV cell line was seen to have the opposite effect using this model and at all time points a significantly greater rate of migration, assessed by changes in resistance following wounding, were observed (Fig. 4b; p < 0.0001 at each time point). An EVOS based conventional scratch wounding assay was also used to assess migration rates of control and knockdown cells over a greater period of time. SOCS-4 knockdown in HaCaT cells was again found to significantly decrease cell migration in comparison to HaCaT pEF6 control, though at much later time points than those seen in the ECIS assays (Fig. 4c). Significant reductions in the migration rate of HaCaT SOCS-4 knockdown cells compared to HaCaT pEF6 cells was observed following 8 hours incubation and continued to the end of the experiment (8 hours, 9 hours, 10 hours p < 0.05, 11 hours, 12 hours, 13 hours p < 0.01). In contrast with the ECIS results, assessment of cell migration in the HECV cell line using this model similarly demonstrated a decreased rate of migration was associated with SOCS-4 knockdown, compared to HECV pEF6 controls and this was again observed in the later stages of the experiment (Fig. 4d), with significant deviations observed from 13 hours onwards (all points p < 0.05). Knockdown of SOCS-4 was also observed to impede the tubule formation capacity of HECV cells seeded onto Matrigel and quantification of total tubule perimeter per field demonstrated a significant decrease in tubule formation compared to the HECV pEF6 control (Fig. 4e; p < 0.01).

**Figure 5:**
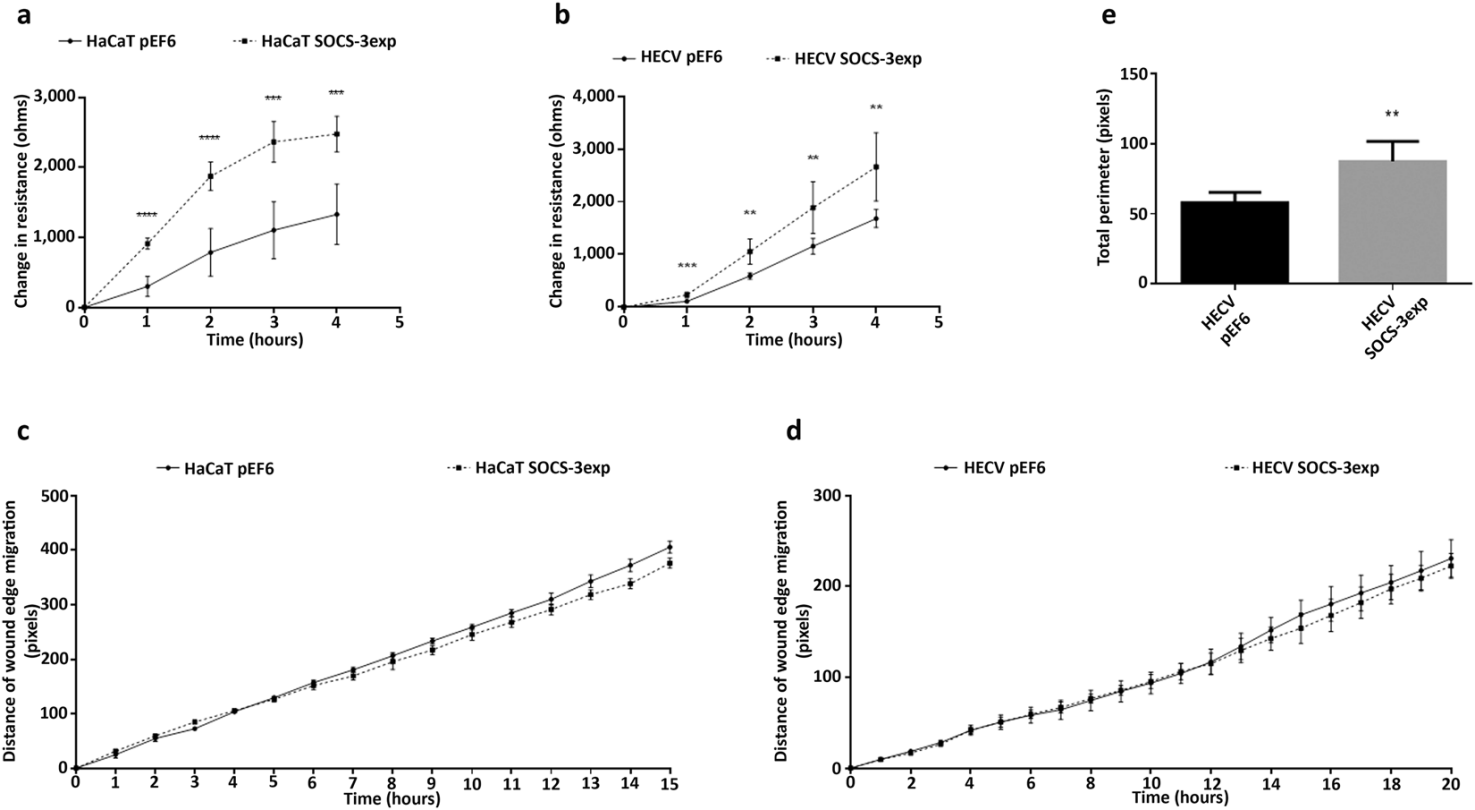
Impact of SOCS-3 over-expression on cellular migration and tubule formation. Cellular migration was assessed using an ECIS model in (**a**) HaCaT and (**b**) HECV cells following SOCS-3 over-expression. Mean change in resistance following monolayer wounding was detected by the ECIS system and was used to quantify cell migration. Cellular migration was also explored using a conventional scratch wound assay, monitored by the EVOS system, in (**c**) HaCaT and (**d**) HECV cells following SOCS-3 over-expression. Angiogenic potential was assessed following SOCS-3 over-expression in HECV cells using a Matrigel tubule formation assay (**e**). Data shown is representative of a minimum of three repeats, error bars represent standard deviation (a/b/e) and standard error of the mean (c/d), ** represents p < 0.01, *** represents p < 0.001 and **** represents p < 0.0001.

ECIS based migration assays demonstrated an enhanced rate of migration following SOCS-3 over-expression in both HaCaT (Fig. 5a; 1, 2 hour p < 0.0001, 3, 4 hour p < 0.001 vs HaCaT pEF6) and HECV cells (Fig. 5b; 1 hour p < 0.001, 2, 3, 4 hour p < 0.01 vs HECV pEF6). In contrast to this, EVOS based scratch wound analysis demonstrated no significant differences between pEF6 control and SOCS-3 over-expression lines in both HaCaT (Fig. 5c) and HECV (Fig. 5d) cells. However, SOCS-3 over-expression was found to significantly enhance tubule perimeter in comparison to HECV pEF6 control cells (Fig. 5e; p < 0.01) indicating that SOCS-3 over-expression enhances the angiogenic potential of HECV cells.

### Impact of SOCS-4 suppression on the responsiveness of HaCaT keratinocytes and HECV endothelial cells to epidermal growth factor and transforming growth factor beta

**Figure 6:**
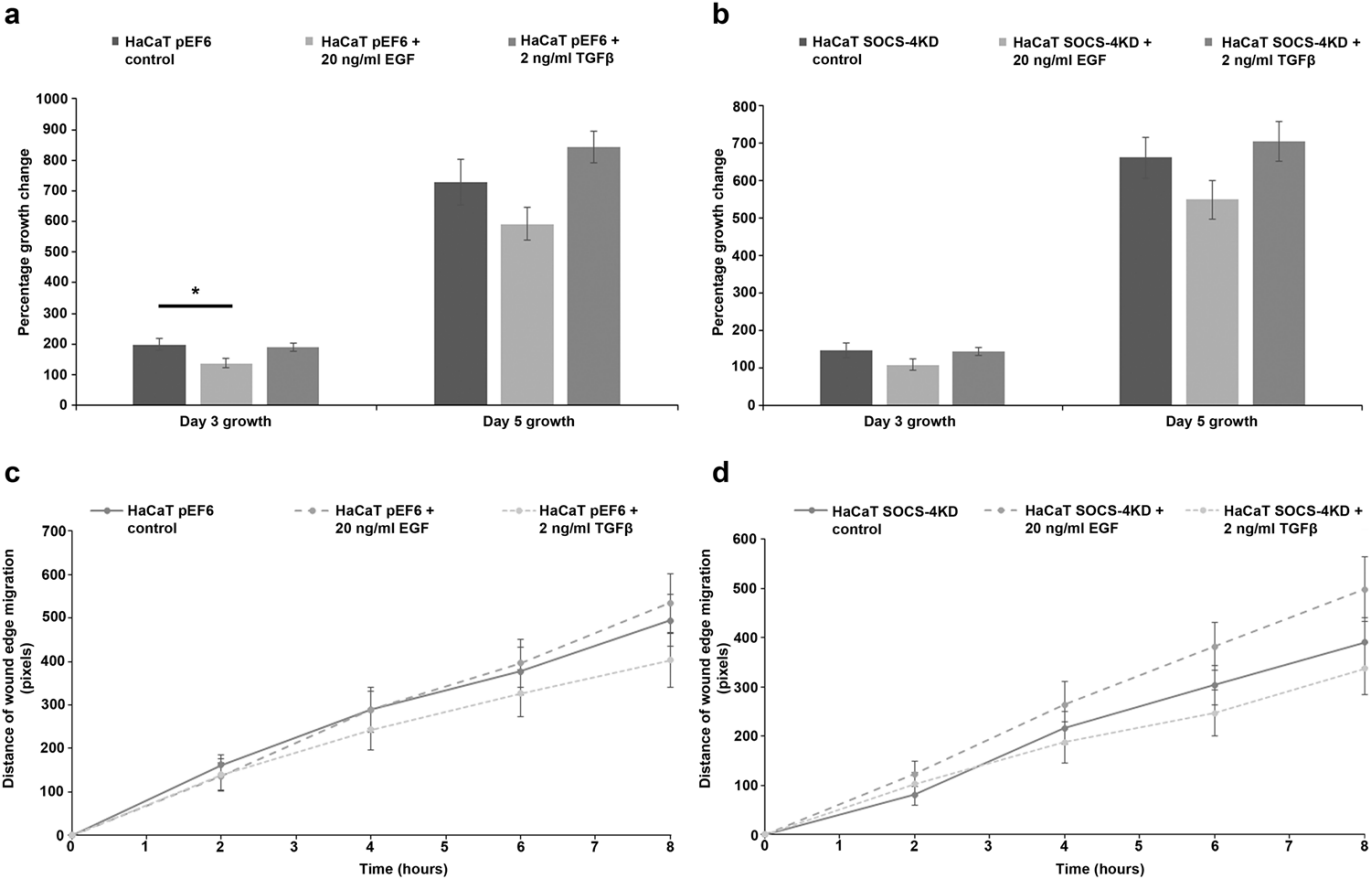
Impact of SOCS-4 suppression on the proliferative and migratory responses of HaCaT keratinocytes to EGF and TGFβ. Proliferative rates of (**a**) HaCaT pEF6 and (**b**) HaCaT SOCS-4 knockdown cells in addition to 20 ng/ml EGF or 2 ng/ml TGFβ were assessed using a MTT growth assay over 3 and 5 days. Cellular migration in response to 20 ng/ml EGF and 2 ng/ml TGFβ was also explored using a conventional scratch wound assay on (**c**) HaCaT pEF6 and (**d**) HaCaT SOCS-4 knockdown cells. Data shown are mean values of a minimum of three independent repeats, error bars represent the standard error of the mean and * represents p < 0.05.

**Figure 7:**
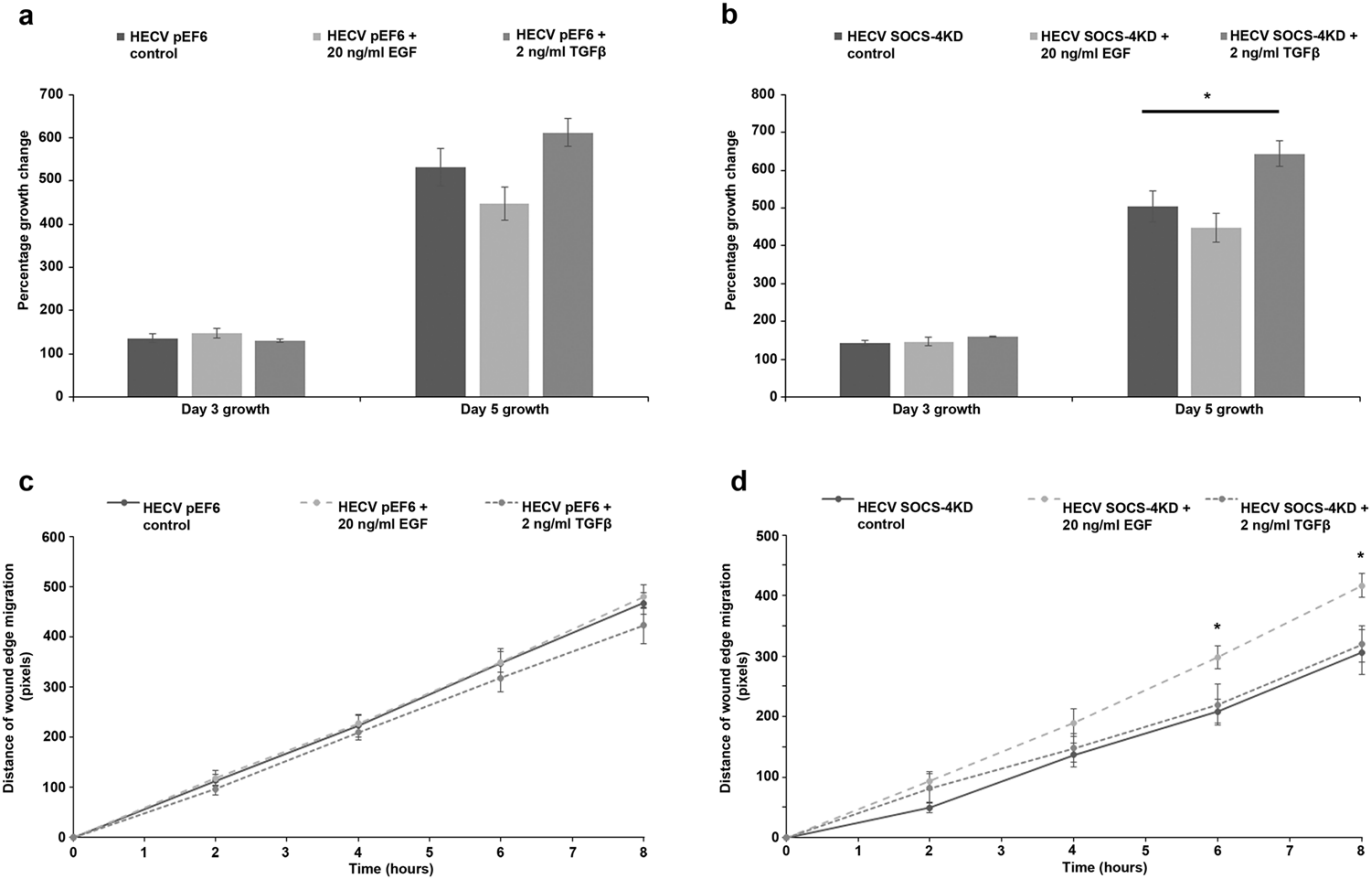
Impact of SOCS-4 suppression on the proliferative and migratory responses of HECV endothelial cells to EGF and TGFβ. Proliferative rates of (**a**) HECV pEF6 and (**b**) HECV SOCS-4 knockdown cells in addition to 20 ng/ml EGF or 2 ng/ml TGFβ were assessed using a MTT growth assay over 3 and 5 days. Cellular migration in response to 20 ng/ml EGF and 2 ng/ml TGFβ was also explored using a conventional scratch wound assay on (**c**) HECV pEF6 and (**d**) HECV SOCS-4 knockdown cells. Data shown are mean values of a minimum of three independent repeats, error bars represent the standard error of the mean and * represents p < 0.05.

In order to gain insight into the significance of SOCS-4 in a more representative wound environment, cellular proliferation and migration assays were repeated, in the presence of 20 ng/ml epidermal growth factor (EGF) or 2 ng/ml transforming growth factor beta (TGFβ), for both HaCaT keratinocytes (Fig. 6) and HECV endothelial cells (Fig. 7). Treatment of HaCaT pEF6 control cells and SOCS-4 knockdown cells with EGF appeared to reduce proliferation rates in comparison to the respective untreated control and this trend was seen to be significant following HaCaT pEF6 treatment with EGF at the early 3 day time point (p < 0.05 vs untreated HaCaT pEF6), though this level of significance was not reached at the later 5 day time point. The addition of TGFβ appeared to bring about a slight enhancement in proliferation in both HaCaT pEF6 and HaCaT SOCS-4 knockdown cells, though this effect was not found to be significant (Fig. 6a,b). Scratch wounding assays (Fig. 6c,d), performed in the presence of each of the growth factors, indicated a small, non-significant decrease in migration in both pEF6 and SOCS-4 knockdown HaCaT cells following treatment with TGFβ. Interestingly, whilst treatment with EGF did very little to influence the migrational rates of pEF6 control HaCaT cells, a clear enhancement was seen following EGF treatment of SOCS-4 knockdown cells, though this did not reach significance. Similar trends were seen within the HECV endothelial cell models. Proliferation of both pEF6 and SOCS-4 knockdown cells was generally unaffected by either treatment at the 3 day time point, though, as with HaCaT cells, a slight, non-significantly decreased proliferative rate was seen following EGF treatment at the 5 day incubation point. Interestingly, TGFβ treatment again enhanced 5 day proliferative rates in both pEF6 and SOCS-4 knockdown cells, though a greater effect of TGFβ was seen following SOCS-4 knockdown (p < 0.05 vs. untreated HECV SOCS-4 knockdown cells) than in the pEF6 control cell line (Fig. 7a,b). TGFβ appeared to slightly reduce HECV pEF6 migration at the later time point, though this was not found to be statistically significant, and had very little impact on the migration rates of SOCS-4 knockdown HECV cells. In keeping with the observed effects of EGF on HaCaT cell migration, treatment of HECV cells with EGF had a differential effect on the migration of pEF6 control and SOCS-4 knockdown cells. Treatment with EGF did not obviously impact on HECV pEF6 cell migration but brought about a significant increase in HECV SOCS-4 knockdown cells at the later 6 hour and 8 hour time points (both p < 0.05 vs. untreated HECV SOCS-4 knockdown cells) (Fig. 7c,d).

### SOCS-4 suppression alters phospho protein expression within HaCaT keratinocytes

**Figure 8:**
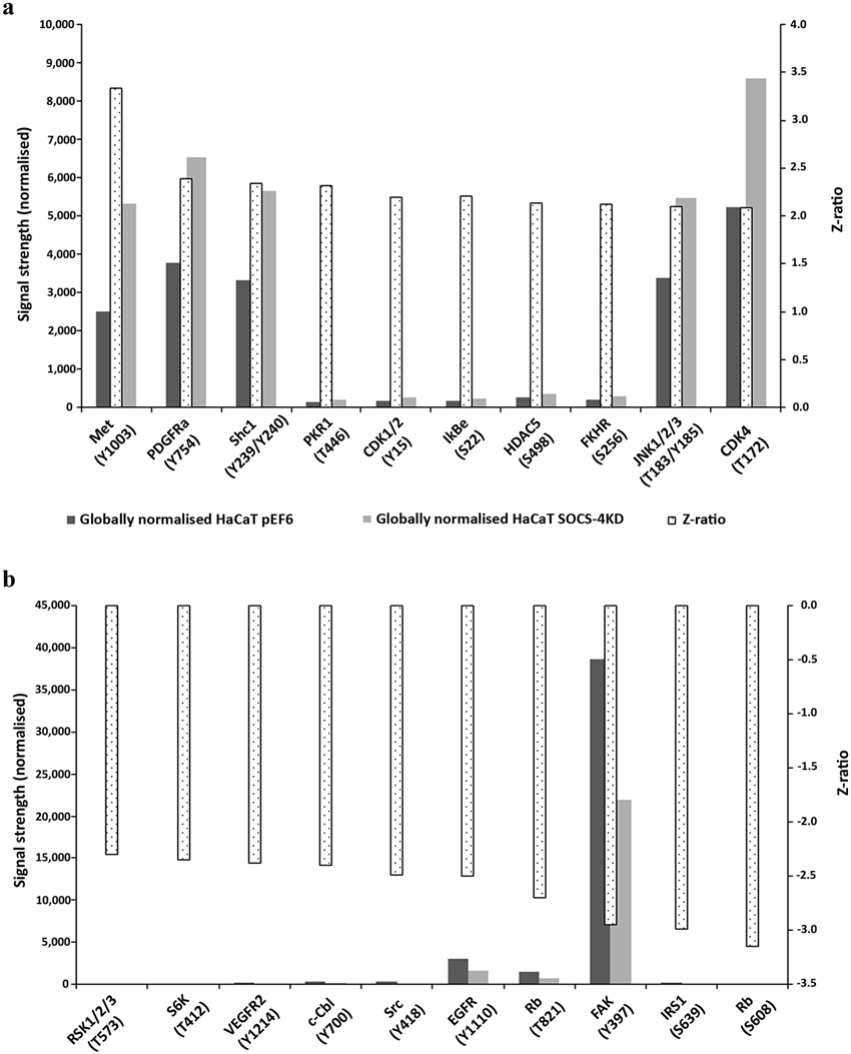
Impact of SOCS-4 suppression on HaCaT phosphorylation status. Summary of Kinexus protein microarray analysis highlighting the ten most highly upregulated (**a**) and down regulated (**b**) phospho proteins within HaCaT keratinocytes following SOCS-4 knockdown.[Met, met proto-oncogene; PDGFRa, platelet derived growth factor receptor alpha; shc1, SH2 domain-containing transforming protein 1; PKR1, Double-stranded RNA-dependent protein-serine kinase; CDK1/2, cyclin-dependent kinase 1/2; IkBe, NFKB inhibitor epsilon; HDAC5, Histone deacetylase 5; FKHR. Forkhead box protein O1; JNK1/2/3, c- Jun N-terminal kinase 1/2/3; CDK4, Cyclin-dependent protein-serine kinase 4; RSK1/2/3, Ribosomal S6 protein-serine kinase 1/2/3; S6K, Ribosomal protein S6 kinase beta-1; VEGFR2, Vascular endothelial growth factor receptor-tyrosine kinase 2; c-Cbl, Signal transduction protein CBL; Src, Src proto-oncogene-encoded protein-tyrosine kinase; EGFR, Epidermal growth factor receptor-tyrosine kinase; FAK, Focal adhesion kinase; IRS1, Insulin receptor substrate 1; Rb, Retinoblastoma-associated protein 1].

In order to explore the implications of targeting SOCS-4 in keratinocytes, a protein microarray was undertaken to explore differences in phospho protein expression patterns in control and SOCS-4 knockdown HaCaT cells. Samples were sent to and processed by Kinexus Bioinformatics (Vancouver, Canada) using a KAM-880 array chip. Analysis of this chip demonstrated a number of differences between the control and SOCS-4 knockdown samples and included a diverse range of phosphorylation events, potentially implicating a regulatory role for SOCS-4 in a wide variety of signalling pathways. Differential expression of phospho protein expression between the control and SOCS-4 knockdown HaCaT cells was assessed through Z-ratio analysis and scaling. Our results indicated that following suppression of SOCS-4 in HaCaT cells the ten most significantly enhanced phosphorylation events were Met Y1003, platelet derived growth factor receptor alpha (PDGFRα) Y754, SH2 domain-containing transforming protein 1 (ShC1) Y239/Y240, double-stranded RNA-dependent protein-serine kinase (PKR1) T446, cyclin-dependent kinase 1/2 (CDK1/2) Y15, NFKB inhibitor epsilon (IkBe) S22, histone deacetylase 5 (HDAC5) S498, Forkhead box protein O1 (FKHR) S256, c- Jun N-terminal kinase 1/2/3 (JNK 1/2/3) T183/Y185 and cyclin-dependent protein-serine kinase 4 (CDK4) T172 (Fig. 8a). Subsequently, the ten most significantly de-phosphorylated events following SOCS-4 suppression in HaCaT cells were, ribosomal S6 protein-serine kinase 1/2/3 (RSK 1/2/3) T573, ribosomal protein S6 kinase beta-1 (S6K) T412, vascular endothelial growth factor receptor-tyrosine kinase 2 (VEGFR2) Y1214, signal transduction protein CBL (c-Cbl) Y700, Src proto-oncogene-encoded protein-tyrosine kinase (Src) Y418, EGFR Y1110, retinoblastoma-associated protein 1 (Rb) T821, focal adhesion kinase (FAK) Y397, insulin receptor substrate 1 (IRS1) (S639) and Rb S608 (Fig. 8b).

## Discussion

During the proliferation and re-epithelialisation phase, the proliferative behaviour and the migratory action of epithelial cells partially determine wound healing speed, thus, the epithelial cell is an essential cell type involved in the wound healing process. Many studies have indicated that SOCS proteins have regulatory roles in epithelial function with some studies, particularly focusing on SOCS-3, indicating a potential association between SOCS and wound healing components^13–15, 20^. Hence, SOCS family members show promise through their capacity to influence epithelial function and cytokine signalling, as regulators of the wound healing process. Previous work within our laboratory has demonstrated an elevated transcript expression of both SOCS-3 and SOCS-4 in non-healing chronic wounds compared to healing chronic wounds^19^. In the human HaCaT keratinocyte and HECV endothelial cell models used in this study, a differential expression pattern of SOCS-1 to -7 was seen. Clear expression of SOCS-2, -4 and -5 were demonstrated whilst weak SOCS-6 expression was also noted. However, no transcript expression was observed for SOCS-1, -3 and -7. Taken together with our previous clinical observations, the current study aimed to further explore the importance of SOCS-3 and SOCS-4 in wound healing, characterising the effect of inducing SOCS-3 or suppressing SOCS-4 expression to explore their role in keratinocyte and endothelial cell function and implications in wound healing.

SOCS-3 has been found to negatively regulate STAT-3 activation^21^ and given that STAT-3 has been shown to mediate epithelial cell apoptosis^22^, SOCS-3 may hold the potential to regulate epithelial cell growth. In addition, studies on keratinocytes and mouse models discovered a regulatory role of SOCS-3 in the proliferation and differentiation of epithelial cells^14^ and observed a correlation between SOCS-3 and exacerbated inflammation^15^, emphasising the importance of SOCS-3 in the chronic wound environment. This is in line with the finding of further investigations on SOCS-3 deficient mouse models in which severe inflammation resulting from STAT-3 hyper-activation was observed, and skin homeostasis indicated to be tightly regulated via the IL-6/STAT-3/SOCS-3 axis^13^. Another study showed that positive SOCS-3 expression was found in the epithelial and endothelial cells adjacent to inflammatory tissue, though in normal human tissues SOCS-3 is predominantly expressed in endothelial cells^23^. Hence, SOCS-3 may have distinct roles in epithelial and endothelial cells during the inflammation stage. Further studies using SOCS-3 deficient mouse models have suggested a potential role for SOCS-3 in leukocyte infiltration^24^, further highlighting its significance during inflammation. In our current study we generated a human HaCaT keratinocyte and a HECV endothelial cell model over-expressing SOCS-3 and confirmed this over-expression at both the transcript and protein level. *In vitro* cell functional assays were conducted on the SOCS-3 over-expression HaCaT and HECV mutant lines. The upregulation of SOCS-3 did not significantly affect the proliferation rate of the endothelial cells over a 5-day incubation period, whereas, a significant decrease in proliferation was seen in SOCS-3 over-expression HaCaT cells compared to the respective control line. This is in agreement with a previous study on a SOCS-3 stable over-expression HaCaT cell model using a different type of vector^20^, though this inhibition was seen much sooner, following 24 hours opposed to 5 days. This may be due to differing SOCS-3 over-expression efficiencies between the studies. Moreover, transgenic mice over-expressing SOCS-3 in keratinocytes, demonstrated impaired wound closure rates, atrophied wound edge margin epithelia and a reduction in proliferating cells^14^, further strengthening the role of SOCS-3 in suppressing keratinocyte proliferation. Taken together these studies imply an inhibitory role for SOCS-3 in keratinocyte proliferation. Cell attachment following HaCaT and HECV SOCS-3 over-expression was also explored using different methodologies. A significantly elevated adherent ability was observed in the SOCS-3 over-expression HaCaT line at the first hour after cell seeding, and remained consistent until the fourth hour in the ECIS based initial adhesion assay, whereas, no statistical difference was seen in the Matrigel adhesion assay. However, regarding HECV, the upregulation of SOCS-3 increased the adhesive ability of this cell line in the Matrigel adhesion assay, whereas, no significant effect of SOCS-3 over-expression on HECV adhesion was found in the ECIS based initial adhesion assay. Such discrepancy between the two methodologies may suggest that SOCS-3 affects keratinocyte and endothelial cell adhesion to different surface matrix via different mechanisms within each cell type or be due to differences between the measurement of resistance change and more conventional methods to quantify adhesion in the Matrigel based assays. However, since the structure and texture of the Matrigel matrix resembles the extracellular matrix (ECM) environment in many tissues and has been extensively recognised, the observations from the Matrigel adhesion assay may indicate a more accurate physiological trend. Similarly, differential results were seen between EVOS based and ECIS based migration assays. SOCS-3 over-expression did not affect keratinocyte or endothelial cell migration in the EVOS based migration assay, whereas significantly elevated migration onto the gold electrode was observed in the ECIS based migration assay. This may again be due to the different cell substratum that the cells migrate across or different sensitivities in the quantification of the migration, whether this be physical distance between two wound fronts or changes in resistance resulting from cells moving onto an electrode. A previous *in vivo* study on a keratinocyte-specific SOCS-3 over-expressing mouse model discovered a severely impaired wound healing phenotype accompanied by an augmented inflammatory response, alteration of the inflammatory chemokine profile and a prolonged presence of neutrophils and macrophages, indicating the detrimental effect of the SOCS-3 on wound healing^15^. Interestingly, no significant change, in terms of migratory rate, was seen in either keratinocyte or endothelial cells following SOCS-3 over-expression from the EVOS migration assay, which seems to be contradictory to the previously reported *in vivo* study. However, the systems tested here are simple *in vitro* assays which may not fully take into account the more complex microenvironment and subsequent cross-talk between cells and a variety of cytokines, growth factors and other contributing factors present in the complex *in vivo* systems. Additionally, since there is little evidence demonstrating the direct *in vitro* effect of SOCS-3 on keratinocyte migration, it is possible that SOCS-3 may be a more significant factor in the context of extra-cellular stimuli. For example Tokumaru *et al*. previously highlighted the importance of SOCS-3 in the phosphorylation of STAT-3 and in hepatocyte growth factor (HGF) induced migration within keratinocytes^25^. SOCS-3 over-expression in HECV endothelial cells resulted in enhanced tubule formation on Matrigel, suggesting a pro-angiogenic influence. Many studies have demonstrated that SOCS-3 plays an inhibitory role in angiogenesis in a variety of pathologies, especially cancer^26^. Interestingly, SOCS-3 has been discovered to be a suppressor of pathological angiogenesis but not a regulator of physiological vascularisation in an investigation on a SOCS-3 deficient mouse model^27^. Thus, it may be that the influence of SOCS-3 on angiogenesis may depend on the particular microenvironmental conditions or particular cell type tested. Hence it is of importance to further explore the angiogenic influence of SOCS-3 in other endothelial cell lines.

Unlike SOCS-3, SOCS-4 is the least investigated molecule within the SOCS family, and there are currently no studies reporting the potential link between SOCS-4 and wound healing. SOCS-4 has been found to be induced by EGF stimulation^28^, and in turn inhibits EGFR-dependent signalling via competing for the docking site with STAT-3 to the phospho-residue on EGFR and through the elevation of EGFR degradation^29^. In addition, the downregulation of SOCS-4 correlates with the elevation of STAT-3 and STAT-6^17^, indicating multiple potential targets of SOCS-4 on the STAT family members. Investigations using a SOCS-4 deficient mouse model and a SOCS-4 knockdown cell model demonstrated the important regulatory role of SOCS-4 in the inflammatory response to influenza and parasite infection, as well as a novel function of SOCS-4 in modulating T cell receptor (TCR) signalling^16, 17^. Moreover, another study hypothesised that SOCS-4 may have a regulatory effect on HIF-1α^18^. Since EGF, STAT-3, STAT-6 and HIF-1α, as well as inflammation, are components involved in the wound healing process, it is feasible that SOCS-4 may also play an important role in regulating wound healing, and this is further highlighted by our previous clinical observations indicating the dysregulation of this molecule between non-healing and healing chronic wounds^19^. To further explore the importance of SOCS-4 we targeted SOCS-4 expression in both human HaCaT keratinocytes and HECV endothelial cells using ribozyme transgenes, confirming successful knockdown at both transcript and protein levels. Characterisation of these mutant lines demonstrated that SOCS-4 did not have any effect on the proliferative rate of these cell types, at least within 5 days. Additionally, HaCaT migration was significantly impaired following SOCS-4 downregulation, and such a trend was consistent in both the ECIS and EVOS migration assays conducted, although the significant difference between the SOCS-4 knockdown and the control line was delayed 8 hours in the EVOS assay in comparison to the ECIS assay. This again may represent differences in the sensitivities of the two methodologies and their quantification of cell migration. Interestingly, the investigation on the migration of SOCS-4 mutant endothelial cells demonstrated contradictory results between the two methodologies, demonstrating a pro-migratory effect in the ECIS based system and an inhibitory effect, similar to that seen in HaCaT cells, in the EVOS based system. This may potentially be due to the different substratum and how SOCS-4 regulates migration across such matrix within the HECV cell line and will require further clarification utilising additional methodologies. The data does however provide novel evidence that SOCS-4 plays an active role in the regulation of migration in these two cell types. A study on a thyroid cancer cell line has suggested that SOCS-4 has an inhibitory effect on migration and that inhibition of SOCS-4 by miR-25 could promote thyroid cancer cell migration^30^. In the current study, particularly in terms of HaCaT keratinocytes, suppression of SOCS−4 inhibited migration, indicating a pro-migratory role for SOCS-4 in this cell line. This may highlight a diverse regulatory role for SOCS-4 in different cellular microenvironments through variant signalling pathways or potentially cell specific functions. Similar to the results obtained for the SOCS-3 over-expression models, contrasting results were seen between the ECIS based and Matrigel adhesion assays. The initial adhesive ability of HaCaT was significantly decreased following the downregulation of SOCS-4 at the first hour following cell seeding and this trend was consistent until the fourth hour in the ECIS based initial adhesion assay, whereas, such cell behaviour was not affected by SOCS-4 knockdown in the Matrigel cell adhesion assay. Moreover, in terms of the HECV cell line, the ECIS based initial adhesion assay displayed a significantly elevated initial adhesive ability of the SOCS-4 knockdown line after cell seeding until the fourth hour, whereas, a contrasting result was observed in the Matrigel cell adhesion assay. As previously discussed, this may relate to differing sensitivities or the differential substratum present in the differing methodologies. Hence our current data may suggest a substratum specific role for SOCS-3 and SOCS-4 and it may be that this relates to some undiscovered role of SOCS-3 and SOCS-4 to regulate cell-matrix adhesion molecules, such as integrins. However, further, intense scientific investigation is required to further explore this theory and fully eluicidate the role of SOCS-3 and SOCS-4 in matrix adhesion. Nevertheless, the data presented, particularly in terms of the HECV endothelial model, strongly suggests that SOCS-4 plays an active role in regulating cell-substrate attachment. We also investigated the impact of SOCS-4 suppression on tubule formation and angiogenic capacity, demonstrating a significant reduction in tubule formation on Matrigel following HECV SOCS-4 knockdown.

To further explore the significance of SOCS-4 in the wound environment and its links to EGFR signalling, we conducted proliferation and migration assays on HaCaT and HECV control and SOCS-4 suppressed cells in the presence of EGF or TGFβ, both cytokines established to be key players in wound healing and dysregulated in chronic wound environments^8, 31^. Previous data has suggested some links between EGF signalling and SOCS-4^28, 29^. In the current study we demonstrated that keratinocytes and endothelial cells with suppressed levels of SOCS-4 appear to have a greater pro-migratory response to EGF and SOCS-4 suppressed endothelial cells have a greater proliferative response to TGFβ. Hence, SOCS-4 appears to be a key player in regulating cellular traits associated with wound healing in response to EGF and TGFβ and may thus be a factor influencing wound response to such growth factors or clinical therapies involving these factors. In keeping with this theory, a wide variety of phospho protein expression changes were also observed, using a protein microarray system, in response to HaCaT SOCS-4 knockdown. The highlighted top 10 up- and down- regulated proteins demonstrate the capacity of SOCS-4 to influence a wide variety of signalling pathways and whilst intense future work will be required to validate and fully elucidate specific mechanisms, these initial observations highlight a number of interesting mechanisms to potentially explain the functional characteristics observed here and further link SOCS-4 to wound healing. Of particular interest was the observation that SOCS-4 suppression decreased Y1110 phosphorylation of EGFR. EGFR signalling has been indicated as an integral pathway involved in wound healing and in keratinocyte function^31^. Similarly, EGFR expression has been previously shown to be reduced in chronic wounds and keratinocytes at the non-healing wound edge were found to be unresponsive to EGF^32^. Phosphorylation of EGFR Y1110 (Y1086) has been proposed to lead to recruitment of additional molecules and progression of downstream signalling^33, 34^. This seems in contrast with our *in vitro* data, demonstrating an enhanced migratory response to EGF. When taken together with the literature it is likely that SOCS-4 plays a complex regulatory role, involving feedback loops, in EGF signalling and how keratinocytes and endothelial cells respond to this growth factor in the wound environment. Further work is required to establish fully the observation of the protein microarray and also to explore such a regulatory relationship in the presence of other factors, such as EGF itself, more representative of a true wound or chronic wound environment.

Other highlighted molecules of interest include the HGF tyrosine kinase receptor Met. HGF/Met signalling has been extensively studied and identified as a key pathway in processes such as tissue repair and cancer progression^35, 36^. Previous work undertaken by our laboratories has explored HGF and Met expression, together with a range of activator and inhibitors, in normal skin and acute as well as chronic wound tissues. Immunohistochemal analysis of these tissues indicated a generally decreased protein expression of Met in chronic wounds compared to acute wounds and this trend was more obvious as you moved further from the wound edge, though q-PCR analysis did not suggest any change in gene expression between these tissue types^37^. The current study identifies a potential mechanism where SOCS-4 suppression could enhance the phosphorylation of Y1003 in Met. This phosphorylation site has been shown to be a binding site for Cbl, resulting in ubiquitination and subsequent internalisation and degradation of the Met receptor, and mutations at this residue has been found to have oncogenic implications^35, 36^. Taken together with our current data, this could suggest a novel role for SOCS-4 in regulating Met turnover within keratinocytes and hence their responsiveness to HGF. Additionally, knockdown of SOCS-4 in HaCaT cells resulted in decreased phosphorylation of focal adhesion kinase (FAK) Y397. Auto phosphorylation of FAK at Y397 is a key event involved in the activation of FAK, occurring in response to numerous stimuli and implicated in a variety of downstream signalling events and functional changes such as enhanced migration^38^. FAK activation has previously been observed to promote keratinocyte migration during wound healing^39^. Additionally, SOCS-4 knockdown also reduced Src Y418 expression, a key activation residue. It has previously been shown that Src activation requires activation of FAK through Y397 phosphorylation and FAK is able to modulate cell migration through interaction of FAK/Src complex and p130Cas^40, 41^. Hence a decrease in both of these phosphorylation events could also account for the decreased rate of migration observed in SOCS-4 knockdown HaCaT cells.

Taken together, the current data has highlighted the potential for SOCS-3 and SOCS-4 to influence a number of cellular traits in HaCaT keratinocytes and HECV endothelial cell lines. Given the importance of these two cell types in the wound healing process the current study provides additional support for the importance of SOCS-3 in wound healing and also novel data demonstrating a potential role for SOCS-4 in this process. Furthermore, our data suggests a role for SOCS-4 in regulating the response of keratinocytes and endothelial cells to factors present in the wound such as EGF and TGFβ. Further scientific study, utilising additional cell lines, primary cultures and more complex multi-cellular environments, is needed to fully elucidate the significance of these SOCS members in wound healing and realise their clinical potential.

## Methods

### Cells and materials

Two cell lines were used for *in vitro* model generation and functional assay investigation in this study. The immmortalised keratinocyte cell line (HaCaT)^42^ was purchased from the German Cancer Research Centre (Heidelberg, Germany). The human HECV endothelial cell line was purchased from Interlab Cell Line Collection (Genova, Italy). Both HaCaT and HECV cell lines were routinely cultured in DMEM/Ham’s F12 with L-Glutamine medium (Sigma-Aldrich, Inc., Dorset, UK), supplemented with antibiotics (Sigma-Aldrich, Inc., Dorset, UK), 10% fetal calf serum (FCS) (Sigma-Aldrich, Inc., Dorset, UK) and incubated at 37.0 °C, 5% CO_2_ and 95% humidity. Recombinant human epidermal growth factor (EGF) and transforming growth factor beta (TGFβ) were purchased from Insight Biotechnology Ltd., (Middlesex, UK) and Biotechne (Abingdon, UK) respectively. EGF was used at a concentration of 20 ng/ml and TGFβ was used at a concentration of 2 ng/ml within the functional assays.

### Total RNA isolation and reverse trascription

RNA was isolated using TRI-reagent (Sigma-Aldrich, Inc., Dorset, UK) under the manufacturer’s instructions. Following isolation, the RNA pellet was resuspended, quantified using a NanoPhotometer^TM^ (IMPLEN; Geneflow Ltd., Lichfield, UK) and standardised before undertaking reverse transcription using the GoScript^TM^ Reverse Transcription System kit (Promega Corporation, Madison, WI, USA) to generate cDNA.

### Polymerase chain reaction (PCR) and agarose gel electrophoresis

Conventional PCR was carried out using GoTaq Green master mix (Promega, Madison, USA) to examine the expression profile of SOCS-1 to -7 in HaCaT and HECV cell lines, and specific primers were designed using Beacon designer software (PREMIER Biosoft International, Palo Alto CA, USA) (Table 1) and synthesised by Sigma-Aldrich, Inc. (Dorset, UK). Reactions were amplified in a 2720 Thermal Cycler (Applied Biosystems, Paisley, UK) before being separated on an agarose gel, stained with SYBRsafe (Life Technologies, Paisley, UK) and visualised using a Syngene gel doc system (U: Genius 3) under blue light.

**Table 1:** Primers/sequences used in the study.

Target gene/Sequence	Forward	Reverse
SOCS-1	5′-GATGGTAGCACACAACCAG	5′-*ACTGAACCTGACCGTACA*GAGGAAGAGGAGGAAGGTT
SOCS-2	5′-GTCAGACAGGATGGTACTGG	5′-CTGGAATTTATATTCTTCCAA
SOCS-3	5′-AAGACCTTCAGCTCCAAGA	5′-GTCTTCCGACAGAGATGCT
SOCS-4	5′-CAGCTGTTCATCCATTGAG	5′-CCATTTGGGTTTGTTTCTT
SOCS-5	5′-AGTCAAAGCCTCTCTTTTCC	5′-*ACTGAACCTGACCGTACA*CATTTTGGGCTAAATCTGA
SOCS-6	5′-CCTTACAGAGGAGCTGAAAA	5′-*ACTGAACCTGACCGTACA*CGAAAAGAAAAGAACCATC
SOCS-7	5′-AGACTAACAGCTGCTCGGAA	5′-CCCACTGATATCATCTAGGAGGC
GAPDH	5′-GGCTGCTTTTAACTCTGGTA	5′-GACTGTGGTCATGAGTCCTT
SOCS-3 (q-PCR)	5′-TCAAGACCTTCAGCTCCA	5′-*ACTGAACCTGACCGTACA*GTCACTGCGCTCCAGTAG
SOCS4 (q-PCR)	5′-GGCAGTGTTTTCCAATAAAG	5′-*ACTGAACCTGACCGTACA*AGGTGGGAAAGGACACTTAT
GAPDH (q-PCR)	5′-CTGAGTACGTCGTGGAGTC	5′-*ACTGAACCTGACCGTACA*CAGAGATGATGACCCTTTTG
SOCS-4 (ribozyme transgene)	5′-CTGCAGTTCACTGATATGAATTTTCCTTTTAGACGCTGATGAGTCCGTGAGGA	5′-ACTAGTCGGCACTCTTCAGGGCTTTTCGTCCTCACGGACT
SOCS-3 (coding sequence amplification)	5′-ATGGTCACCCACAGCAAGTT	5′-TTAAAGCGGGGCATC

Z Sequence represented by ‘*ACTGAACCTGACCGTACA*’.

### Quantitative Polymerase Chain Reaction (q-PCR)

q-PCR was used to verify transcript knockdown or over-expression. This methodology has been previously described and utilised in our laboratory^43^. In brief, primers were designed specifically to SOCS-3 and SOCS-4 (Table 1). To the reverse primer of each pair a Z sequence (ACTGAACCTGACCGTACA) was added in order to allow incorporation of the FAM tagged Uniprimer probe (Intergen Inc., New York, USA) and subsequent fluorescent detection. An internal standard was run alongside the genes of interest and used to generate a standard curve, allowing relative transcript copy numbers to be determined. Sample reactions, including forward primer, reverse Z-sequence tagged primer, Uniprimer probe, cDNA, molecular biology grade water and PrecisionFAST 2X q-PCR Mastermix (PrimerDesign, Eastleigh, UK) were amplified and detected using a StepOne plus (Life Technologies, Paisley, UK) q-PCR system. Target gene expression was further normalised based on the detection of the GAPDH housekeeping gene.

### Generation of SOCS-4 knockdown and SOCS-3 over-expression models

Ribozyme transgenes were specifically designed based on the predicted secondary structure of human SOCS-4 generated using Zukers RNA Mfold programme^44^ and synthesised using touchdown PCR combined with REDTaq® Ready Mix^TM^ PCR Reaction Mix (Sigma-Aldrich, Dorset, UK). Ribozyme sequences used in the study are outlined in Table 1.

A high fidelity enzyme mix kit (Fisher Scientific UK, Loughborough, UK) and specific primer pairs (Table 1) were used to amplify the coding sequence of human SOCS-3 from the MRC-5 normal lung fibroblast cell line. The amplified PCR product was then separated by gel electrophoresis, and the gel was excised at the predicted size of SOCS-3 coding sequence before purification using a GenElute^TM^ Gel Extraction Kit (Sigma-Aldrich, Dorset, UK) and Sanger sequencing of the extract (Source Bioscience, Nottingham, UK) was undertaken to verify identity.

Generated over-expression or ribozyme transgene sequences were subsequently cloned into the pEF6/V5-His TOPO® TA expression system (Life Technologies, Paisley, UK) to generate SOCS-3 over-expression or SOCS-4 knockdown plasmids in accordance with the manufacturer’s guidelines. These plasmids were used to transfect HaCaT and HECV wild type cells using electroporation. Following transfection cells underwent a selective period (media containing 5 µg/ml blasticidin) before being transferred to and cultured routinely in maintenance medium (media containing with 0.5 µg/ml blasticidin).

### Cellular lysis and protein extraction

Upon reaching sufficient confluency, cells were washed with ice cold sterile PBS and scraped from the flask using a sterile cell scraper in sterile PBS. The cell suspension was centrifuged at 2500 rpm for 10 minutes before lysing the pellet in lysis buffer and placing on a rotating wheel (Wolf laboratories, York, UK) at 4 °C for 45 minutes. Subsequently, the lysate was centrifuged for 15 minutes at 13,000 rpm and the supernatant retained and quantified using a Bio-Rad *DC* ^*TM*^ protein assay kit (Bio-Rad, Laboratories, Hemel-Hempstead, UK). The concentration of the protein samples were then standardised to 2 mg/ml, diluted in 2x Lamelli sample buffer (Sigma-Aldrich, Dorset, UK) and boiled at 100 °C for 5 minutes before being stored at −20 °C for further use.

### Tris-glycine sodium dodecyl sulphate polyacrylamide gel electrophoresis (SDS-PAGE) and western blotting

Tris-glycine SDS-PAGE was undertaken using an OmniPAGE VS10DSYS vertical electrophoresis system (OmniPAGE, Cleaver Scientific Ltd., Rugby, UK) to separate proteins before wet transfer to a PVDF membrane (EMD Millipore Corporation, Billerica, MA, USA).

Once complete, the membrane was incubated in blocking solution (5% low fat milk and 0.1% Tween 20 in TBS) for at least 1 hour at room temperature. The membrane was then probed with primary antibody (SOCS-4, R & D systems, Abingdon, UK; SOCS-3, Abcam, Cambridge, UK; GAPDH, Insight Biotechnology Ltd., Middlesex, UK) at 4 °C overnight, subjected to washes and incubated with a horseradish peroxidase (HRP) conjugated secondary antibody (Anti-mouse or Anti-goat, Sigma-Aldrich, Dorset, UK) for 2 hours before washing and protein band detection using a EZ-ECL Chemiluminescent Detection Kit (Biological industries, Kibbutz Beit-Haemek, Israel) and visualisation in a G:BOX Chemi XRQ imaging system (Syngene, Cambridge, UK).

### Electric cell-substrate impedance sensing (ECIS) based initial adhesion and migration assay

Electric cell-substrate impedance sensing (ECIS) instruments (Applied Biophysics Inc., NJ, USA) were applied to investigate cellular behaviour based on the impedance parameter detected from gold electrodes coated on the bottom of a 96-well array (Applied Biophysics Inc., NJ, USA). This method has been previously described^43^. In brief, prior to cell seeding, ECIS arrays containing growth medium were stabilised using the stabilisation function within the system and washed. Cells were seeded at an appropriate density before the 96-well array was equipped in the incubated array station and changes in resistance/impedance measured over the course of the experiment. The first four hours of data was analysed for initial attachment and spreading. Once the resistance curve reached plateau electrical wounding was applied for 20 seconds at 3000μA and 60,000 Hz, to generate a wound in the monolayer before recording the change in resistance following recovery of the monolayer to analyse cellular migration over a four hour period.

### *In vitro* thiazolyl blue tetrazolium bromide (MTT) cell proliferation assay

Cell growth was assessed using a MTT cell proliferation assay. Cells were trypsinised, counted and seeded at a density of 2,000 cells/well. Plates were prepared in triplicate and incubated for 24 (1 day), 72 (3 days) and 120 hours (5 days) at 37.0 °C, 5% CO_2_ and 95% humidity. Following appropriate incubation a working concentration of 0.5 mg/ml MTT was added and the plates were incubated for a further 4 hours. Subsequently, the medium was aspirated and 200 µl of dimethyl sulphoxide (DMSO) was added and incubated for 10 minutes. The solution was gently mixed before reading the absorbance of each well at 540 nm using an ELx800 plate reading spectrophotometer (Bio-Tek, Wolf laboratories, York, UK). The cell number in each well was determined through the absorbance and a percentage increase at each time point was calculated in reference to the day 1 plate.

### *In vitro* cell adhesion assay

Cell adhesion assays were used to examine cellular adhesion to Matrigel matrix (Corning Incorporated, Flintshire, UK). Five micrograms of Matrigel was pre-dried into the wells of a 96 well plate before rehydration and cell seeding at a density of 40,000 cells/well. Following seeding, the plates were incubated for 45 minutes before aspirating the medium and washing with PBS. Two differing methods were subsequently applied to quantify adhesive cells. In the case of SOCS-4 knockdown models, cells were fixed in 4% formalin for a minimum of 10 minutes before washing in water and staining using 0.5% crystal violet (Sigma-Aldrich, Dorset, UK). This was subsequently rinsed off the plate which was allowed to dry before capturing images and quantifying adherent cell numbers. In the case of SOCS-3 over-expression cells, following washing the plate was incubated with 0.5 mg/ml MTT for 4 hours. Subsequently, the solution was aspirated and 200 µl of DMSO added and incubated for 10 minutes. Once complete, this solution was gently mixed before reading the absorbance at 540 nm in an ELx800 plate reading spectrophotometer (Bio-Tek, Wolf Laboratories, York, UK). Initial attached cell number in each well was determined by the absorbance.

### *In vitro* cell migration assay (wound healing assay)

Conventional wound healing scratch assays were also used to explore cellular migration rates and were undertaken using an EVOS® FL Auto Imaging System (Life Technologies, CA, USA) equipped with an EVOS® Onstage Incubator (Life Technologies, CA, USA). Cells were prepared in a 24-well plate and grown to form a monolayer before inducing a wound by scratching a 200 µl pipette tip through the monolayer. Following induction of the wound, the monolayer was washed, fresh medium added and cells allowed to recover for 10 minutes before placing the plate in an EVOS® Onstage Incubator pre-set to 37 °C, 5% CO_2_ and 80% humidity. Image captures were programmed to three separate areas of each wound and each location was imaged at 1 hour intervals, for up to 20 hours. Wound healing scratch assay experiments involving treatments were undertaken using conventional image capture methodologies using an inverted microscope over 2 hour time points for an 8 hour period. Images of the scratch wound were taken at the same area over the time course and multiple regions of wound closure were quantified within this area. For both methodologies, the distance between the two wound edges was measured using Image J, in pixels, and the distance change at every time interval was calculated through subtraction of the distance between each wound edge at each time point from the wound edge distance at time zero.

### Tubule formation assay

A Matrigel tubule formation assay was used to assess the angiogenic potential of human endothelial cells following SOCS-3 and SOCS-4 manipulation. This methodology was adapted from a previously described method^45^. In brief, 50 µl of Matrigel matrix was gently pipetted into the bottom of each well in 96-well plate and incubated until polymerisation had occurred. Subsequently, cells were seeded onto the gel at a density of 40,000 cells/well and the plate was incubated at 37 °C, 5% CO_2_ and 95% humidity. The plate was removed from the incubator and images were captured using a Leica DMi1 microscope equipped with a MC120 HD camera and Leica Application Suite version 3.0.0 software (Leica Microsystems, Milton Keynes, UK) at the 10^th^ hour. The total perimeter of each formed tubule in every image was measured using Image J.

### Kinexus protein microarray

Cells were grown to approximately 80% confluence in T75 tissue culture flasks. Cells were subsequently washed in PBS, scraped from the flasks using a sterile cell scraper and centrifuged to obtain a pellet. Subsequently the pellet was lysed in lysis buffer, transferred to a 1.5 ml eppendorf and placed on a rotating wheel for 40 minutes at 4 °C. Following this, insolubles were removed through centrifugation at 13,000 rpm and the supernatant separated and quantified using a Fluorescamine (Sigma-Aldrich, Dorset, UK) based fluorescent assay, measured using a GloMax – Multi Microplate Multimode Reader (Promega Biosystems Inc., Sunnyvale, CA, USA). Samples were subsequently standardised and sent to Kinexus Bioinformatics, Vancouver, Canada, for protein microarray analysis using a KAM-880 array. Comparisons between HaCaT pEF6 control protein and HaCaT SOCS-4 knockdown protein were drawn to yield percentage change from control (%CFC) and Z-ratio values.

### Statistical analysis

Statistical analysis was undertaken using the SigmaPlot 11 and Graphpad Prism 6 statistical software packages. Data was analysed using t-test or Mann Whitney test, depending on data parameters. Internal replicates were set up within each experiment and each experiment was repeated independently at least three times. Values of p < 0.05 (*), p < 0.01 (**), p < 0.001 (***) and p < 0.0001 (****) were regarded as statistically significant.

### Data availability statement

The data supporting the findings of this study are available from the corresponding author on reasonable request.

## Electronic supplementary material

Supplementary Information
