# Supplementary material for: In vitro significance of SOCS-3 and SOCS-4 and potential mechanistic links to wound healing
Source: Sci Rep. 2017 Jul 27;7:6715. doi: 10.1038/s41598-017-06886-6 (PMC5532239; doi:10.1038/s41598-017-06886-6)
Supplement: Supplementary file 1 — Supplementary Information [file 41598_2017_6886_MOESM1_ESM.pdf]

Supplementary information for SREP-17-11580A

In vitro significance of SOCS-3 and SOCS-4 and potential mechanistic links to wound healing

Yi Feng[1#], Andrew J. Sanders[1#], Liam D. Morgan[1], Sioned Owen[1], Fiona Ruge[1,2], Keith G. Harding[2], *Wen G. Jiang[1]

[1]Cardiff China Medical Research Collaborative and [2]Wound Healing Research Unit, Cardiff University School of Medicine, Cardiff University, Cardiff, CF14 4XN, UK, jiangw@cf.ac.uk

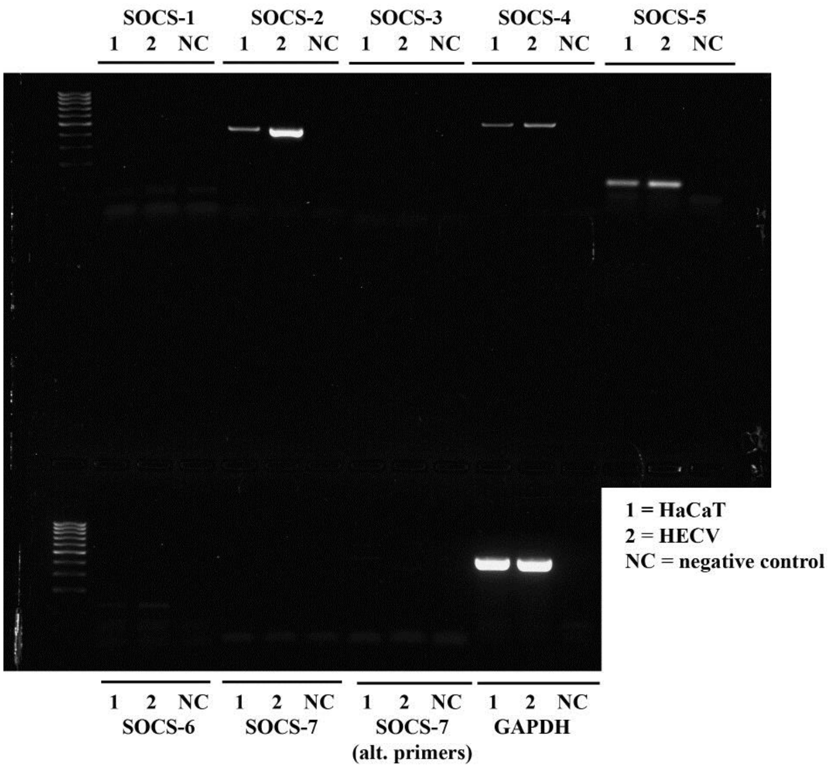

Full gel profile of composite used in figure 1a

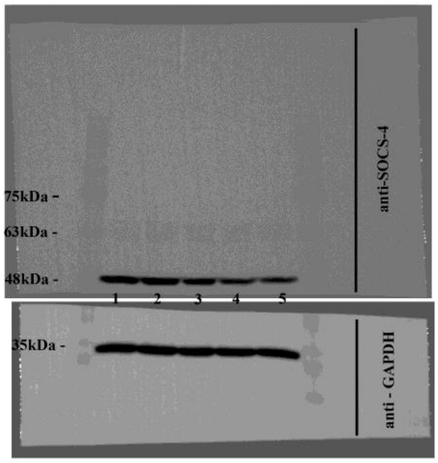

1 = HaCaT pEF6
2 = HaCaT SOCS-4 ribovyme 1-2
3 = HaCaT SOCS-4 ribozyme 1-4
4 = HaCaT SOCS-4 ribozyme 2-1 (used in study)
5 = HaCaT SOCS-4 ribozyme 2-2

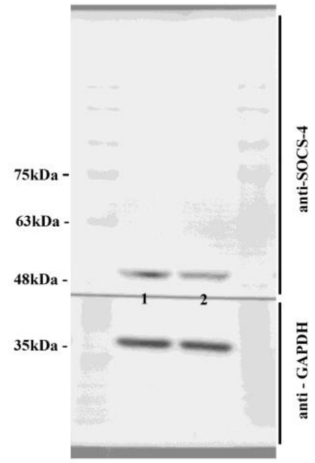

1 = HECV pEF6
2 = HECV ribozyme 2-1 (used in study)

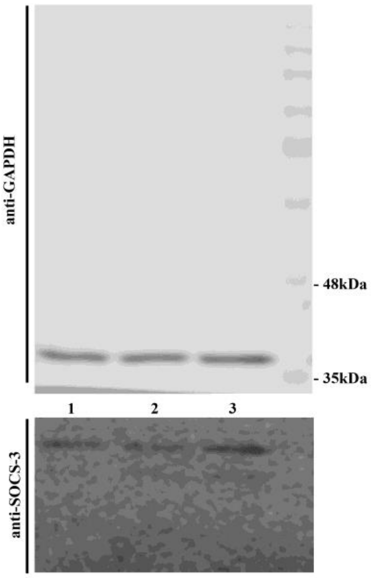

1 = HaCaT pEF6
2 = HaCaT SOCS-3 exp1
3 = HaCaT SOCS-3 exp2 (used in study)

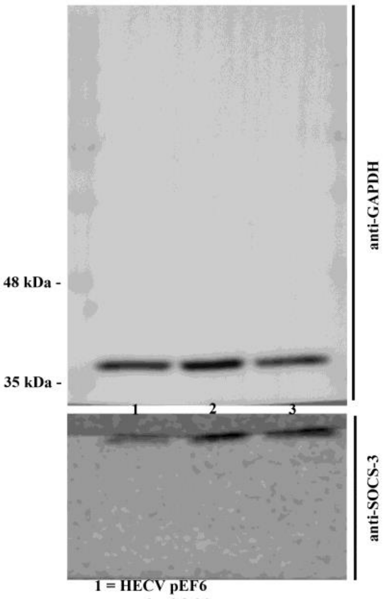

1 = HECV pEF6
2 = HECV SOCS-3 exp1
3 = HECV SOCS-3 exp2 (used in study)

Full length blots used in composites in figure 1d,e,h,i
